# AI-assisted thematic synthesis of existing neurological core outcome sets: A descriptive reference framework (COS-Neuro)

**DOI:** 10.1371/journal.pone.0333864

**Published:** 2026-06-22

**Authors:** Shyun Ping Tiong, Xiaoyu Yang, Alvaro Yanez Touzet, Christopher Paul Millward, Carl M. Zipser, Lindsay Tetreault, Ali Gharooni, Benjamin M Davies

**Affiliations:** 1 Norfolk and Norwich University Hospitals NHS Foundation Trust, Norwich, United Kingdom; 2 University of East Anglia, Norwich Medical School, Norwich, England; 3 Department of Surgery and Cancer, Imperial College London, London, United Kingdom; 4 Neuroscience Research Team, Imperial College Healthcare NHS Trust, London, United Kingdom; 5 University of Manchester, Manchester, United Kingdom; 6 University of Liverpool, Liverpool, United Kingdom; 7 Spinal Cord Injury Center and Department of Neurology and Neurophysiology, Balgrist University Hospital, University of Zurich, Zurich, Switzerland; 8 Department of Neurology, New York University Langone Health, New York, United States of America; 9 Division of Neurosurgery, Department of Clinical Neurosciences, University of Cambridge, Cambridge, United Kingdom; 10 Myelopathy.org, Cambridge, United Kingdom; 11 School of Surgery, Northwest Deanery, United Kingdom; 12 Department of Neurosurgery, Cambridge University Hospital, Cambridge, United Kingdom; Banner Alzheimer’s Institute, UNITED STATES OF AMERICA

## Abstract

**Background:**

Neurological disorders affect approximately 3 billion people globally, yet clinical trial success is often hindered by poorly selected outcome measures, impacting trial design, compliance, and interpretation. Over the past 25 years, Core Outcome Sets (COS) have emerged as standardized tools to enhance outcome selection, ensuring comparability across studies and reflecting the priorities of both researchers and patients. Despite the success of COS initiatives in other fields, their development in neurology remains limited, leaving many trialists without disease-specific guidance.

**Objectives:**

This study aimed to conduct an AI-assisted thematic synthesis of outcome domains from existing COS, with the goal of identifying structural patterns common to these sets and generating descriptive reference framework to inform future COS development.

**Methods:**

COS-Neuro was developed using AI-assisted thematic framework analysis, complemented by expert review. A modified five-step thematic analysis was conducted without pre-determined codes: 1. Dataset Gathering – Data was collected from the COMET database, and COS domains for neurological disorders were coded. 2. Prompt Design & Testing – Large language models (LLMs), including ChatGPT 3.5, Google Gemini 1.5 Flash and Meta Llama-2-70b, were trialled, and prompts refined based on their outputs. 3. Thematic Analysis – LLMs categorised domains into core areas. 4. Human Refinement – Experts reviewed LLM-generated core areas and selected those most appropriate for further interpretation. 5. Clinical Validation – Experts validated the domains, core areas, and concepts. This approach integrated AI with expert oversight to develop an AI-descriptive thematic map of existing neurological COS.

**Results:**

Utilising LLMs, particularly ChatGPT, an AI-assisted conceptual framework synthesising existing neurological COS was developed based on the analysis of 112 published COS. Through adaptation of the OMERACT model, the final framework comprised four overarching concepts, 13 core areas, and 75 domains identified through expert consensus.

**Conclusion:**

COS-Neuro provides a preliminary AI-assisted descriptive synthesis of existing neurological COS, organised using the OMERACT Filter 2.1 as a structural reference. This hypothesis-generating framework may serve as a foundational resource for future COS research and trial design, particularly in areas where no disease-specific COS exists. However, it requires prospective validation through disease-specific, multi-stakeholder consensus processes before clinical application. COS-Neuro also demonstrates the feasibility of AI-assisted thematic synthesis in this context and warrants evaluation in other specialties.

## Introduction

Neurological disorders are the leading cause of illness and disability worldwide, affecting an estimated three billion people [1]. Translational research is critical to addressing these unmet needs [2]. As of March 2025, more than 10,800 clinical trials in neurological disorders were ongoing, accounting for 21.5% total clinical trials registered on clinicaltrials.gov.

While trial success depends on multiple factors, the choice of trial outcome measure is fundamental. Outcome measures inform sample size calculation, influence participant compliance and attrition, and ultimately determine how trial results are interpreted; put simply, if outcome measures are poorly chosen, a trial may reach incorrect conclusion or struggle to influence clinical practice [3–5].

An important initiative supporting outcome selection in clinical trials over the last 25 years has been the development of standardised outcome sets, known as Core Outcome Sets (COS) [6]. COS are a list of critical disease features that should be measured for a particular disease or condition. Primarily designed to facilitate research synthesis by ensuring studies can be compared without bias, COS developed through broad stakeholder engagement – including individual lived experience – also help ensure that studies measure a disease comprehensively and capture outcomes that matter most to patients [7]. Initiatives such as COMET (Core Outcome Measures in Effectiveness Trials) and OMERACT (Outcome Measures in Rheumatology) have significantly advanced COS development and refined best practice for their creation. A recent impact assessment of the COS for rheumatoid arthritis, one of the earliest examples, highlights the potential benefits for trialists, with translational success in the field correlating with its adoption rate, which now stands at 82% [8].

The potential for COS to support translational science is clear; however, their development and implementation remain time consuming and costly [9]. To date, only 55 established COS (as of August 2024) have been developed for neurological diseases, including cases where multiple COS exist for a single condition within the COMET database. Consequently, many neurological trialists lack disease-specific COS as a reference when designing their trial [10]. Although frameworks such as the OMERACT Filter provide conceptual guidance, they remain largely specific rheumatology and may not fully address the unique complexities of neurological research.

A review of neurological COS reveals many common themes, leading us to hypothesize that a descriptive thematic map might be derivable from existing COS in neurological disorders. Unlike the OMERACT Filter, such sector-specific guidance could offer a preliminary reference point for trialists and COS developers, though its utility in conditions without established COS remains to be validated.

This study (COS-Neuro) describes an AI-assisted thematic synthesis of domains from existing neurological COS, with the aim of identifying structural patterns common to these sets and producing a reference framework. While COS-Neuro synthesises a comprehensive body of neurological COS based on existing COS, its framework should be regarded as a preliminary synthesis bounded by the scope of currently available neurological COS, rather than a validated framework applicable across all conditions.

## Methods

This study employed a mixed-method design, combining semi-automated AI-based analysis with expert review to propose cross-cutting themes for neurological disorders.

Thematic analysis (TA) is a qualitative research method for identifying and analysing patterns or themes within a dataset through process such as data familiarisation and coding [11]. AI is increasingly transforming qualitative research due to its cognitive ability in data processing, coding and pattern recognition. It has been shown to effectively assist in process of thematic analysis [12]. Large Language Models (LLMs) are advanced AI models trained on massive text datasets, enabling them to comprehend contextual data. Coupled with natural language processing (NLP), LLMs can generate contextually relevant outputs and identify themes by analysing patterns across diverse domains [13]. With the appropriate prompt, LLMs can enhance data visualisation, analysis and summarisation of scientific text [14,15].

This COS-Neuro project was developed using thematic framework analysis [16]. Themes were generated through AI-assisted identification and interpretation of data, followed by review and refinement by clinicians with experience and expertise in neurology and neurosurgery, COS and / or clinical trials.

**Fig 1:**
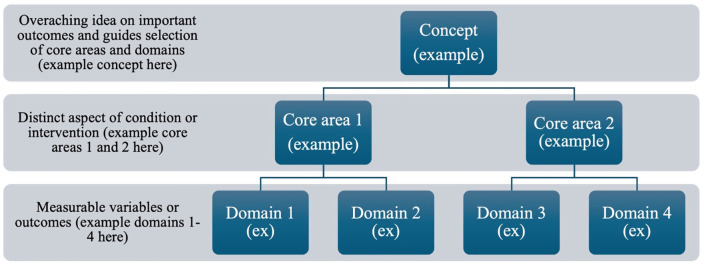
Hierarchical diagram for COS-Neuro development (adapted from OMERACT Filter 2.1).

Several initiatives have contributed significantly to the development and dissemination of COS, including OMERACT [17] and COMET initiative [18]. Amongst the methodological resources created by OMERACT is the OMERACT Filter, which was updated in 2019 to Version 2.1. The filter aims to support COS developers in ensuring content validity by categorising the universe of measurable healthy-related concepts [19]. The framework defines a measurement ‘concept’, such as Pathophysiology and Impact, which maps to a ‘Core Area’, such as Death, Life Impact, Societal/Resource Use, Manifestation and Abnormalities (Fig 1).

The figure illustrates the three-tiered structure underpinning the COS-Neuro framework. At the highest level, *concept* represent overarching domains that define key aspects of outcome assessment. These concepts are subdivided into *core areas*, which capture distinct dimensions of disease or intervention. Each core area is further operationalised through *domains*, representing specific, measurable outcomes.

Using OMERACT Filter 2.1 as a structural scaffold, the primary goal of COS-Neuro was to combine AI-generated thematic categories and expert reviews to create a descriptive reference framework for neurological disorders. The development of COS-Neuro was pragmatic and intentionally provisional, prioritising efficiency amid uncertainty whether the approach was feasible. Therefore, the process relied on a small working group, with consideration of balance in expertise and geographic representation.

A panel of six clinicians / researchers participated in the expert review and consensus process for COS-Neuro. Experts were purposively selected based on:

- Clinical experience in neurology or neurosurgery.
- Prior involvement in COS development or outcome research.
- Academic publications related to neurological disorders.

The panel included neurologists, neurosurgeons, and clinical researchers from the United Kingdom, Switzerland, and the United States. Table 1 summarises their professional background and relevant expertise.

**Table 1:** Background and Expertise of Panels Participating in Consensus Process.

Clinician Initials	Specialty	Institution	COS experience	Years of clinical experience
XY	Neurosurgery	Imperial College London, UK	COS in DCM, trials	7
CMP	Neurosurgery	University of Liverpool	COS in Meningioma	11
CMZ	Neurology	University of Zurich, Switzerland	COS in DCM, Research in Acute Spinal Cord Injury	12
LT	Neurology	New York University Langone Health, US	COS in DCM, Research in Neuromuscular Medicine	5
AG	Neurosurgery	University of Cambridge, UK	COS in DCM	9
BD	Neurosurgery	University of Cambridge, UK	COS in DCM	12

The consensus process followed a modified nominal group technique. After initial AI-assisted thematic analysis, experts reviewed the proposed core areas and domains in an iterative process:

1. Round 1: Experts individually reviewed the initial AI-derived domains and provided written feedback via email.
2. Round 2: A virtual meeting was held where experts discussed discrepancies, suggested domain refinements, and proposed new domains or core areas.
3. Round 3: A revised list was circulated for final approval. Consensus was defined as ≥75% agreement on inclusion of domains or thematic groupings.

Disagreements were resolved through discussion during meetings until consensus was achieved. Detailed records of feedback and revisions were maintained to ensure transparency.

Clinical validation thus integrated AI output with human expertise to establish a robust, clinically meaningful COS framework for neurological disorders. It is important to note that COS-Neuro was designed as a preliminary, hypothesis-generating exercise. The expert panel provided oversight and validation of AI outputs but this process does not constitute a formal multi-stakeholder consensus in accordance with established COS development standards.

A modified five-step thematic analysis framework was used, where the themes were created without any predetermined codes [20].

### Step 1 – Dataset gathering

Data were extracted from the COMET database, which stored studies relevant to development of COS for use in clinical trials [21]. The term ‘neurology’ was used in the search, and only published or ongoing studies were included in the dataset at the time of data collection. The analytic dataset was fixed at August 2022 to ensure reproducibility. The focus was on neurology, neurosurgery, and stroke as main specialties. The data were extracted by CPM.

The COS were selected by reviewers with expertise in neurological disorders (neurosurgeons and neurologists) with inclusion and exclusion criteria outlined in Table 2. Each study was coded based on the COMET ID to facilitate cross-checking of domains by different reviewers. Established core outcomes for each neurological disorder were longlisted in Microsoft Excel (Version 16.91) for analysis.

**Table 2:** Inclusion and exclusion criteria for selecting existing Core Outcome Sets (COS).

Inclusion criteria	Exclusion criteria
Neurology-related studies	Unpublished COS
Brain injury	Withdrawn COS
Spinal cord disorders	Ongoing COS
Dementia	
Neurodegenerative diseases	
Brain injury	
Brain tumour	
Functional neurological disorder	

### Step 2 – Prompt design and trial

Three LLMs - CHATGPT 3.5, Google Gemini 1.5 Flash, and Meta Llama 2 70b - were used to analyse the longlist of domains. These models were selected due to their accessibility and advanced developed in the field of AI [22]:

ChatGPT builds on Transformer Neural Network architecture, excelling in a wide range of NLP Task. It predicts the next word based on the contextual learning from pre-training and is capable of identifying statistical patterns and structural characteristics of human language. Google Gemini is known for outperforming human respondents in Massive Multitask Language Understanding (MMLU) tasks, including standardised exams and image recognition, through training on multimodal and multilingual datasets. Llama encompasses pre-trained and fine-tuned generative text models ranging from 7 billion to 70 billion parameter [22].

Prompt engineering was adapted to design prompts to guide thematic analysis using LLM. This involved optimising prompts to achieve desirable outcomes from generative model. In this study, instructions + input subgroup approach [23] was used to construct prompts across all three models.

Role prompting was employed as an effective strategy to initiate the task for a specialised topic [24]. Prompts were structured on included a background information, specific instructions, and examples to minimise deviation from expected outcome.

For this project, prompts were framed to assign the LLM the role as medical researcher (a trialists), with instructions to perform thematic analysis on core outcome measurements and propose suitable cross cutting themes based on given domains.

A stop criterion for further prompt generation was defined as thematic saturation – when all the major domains of neurological disorder outcomes were adequately represented in the core set at clinician’s consensus, and additional analysis no longer yielded new insights [25].

Response across the three LLMs was reviewed, with feedback incorporated for further refinement. Three large language models (LLMs) were evaluated: ChatGPT-3.5 (OpenAI), Gemini 1.5 Flash (Google), and Llama-2-70B (Meta). Models were accessed via publicly available web interfaces. Default generation parameters were used. Each prompt was entered into a new session to minimise carry-over context effects unless iterative refinement was required.

Prompt development followed an iterative refinement process. Initial prompts were drafted by two investigators (SPT, XY) and tested across all three models. Outputs were reviewed by the listed expert panel participated in the consensus process and modified to improve clarity, reduce ambiguity, and align with OMERACT conceptual structure.

To assess output stability, selected prompts were repeated under identical conditions. Variability in thematic groupings was assessed qualitatively by two clinicians. Final model selection was based on ability to process the full dataset, conceptual coherence, and clinical interpretability of output. All AI-generated outputs were exported verbatim and archived. Reviewers (SPT, XY, AYT, BMD) independently annotated outputs, identifying domains requiring consolidation, reclassification or exclusion to further optimise the prompts.

### Step 3 – Thematic analysis

An in-depth inductive thematic analysis was conducted to generate outcomes domains. Data were analysed inductively using a combination of LLMs outputs and clinical expertise to identify and interpret outcomes relevant for patients [26], and to assess the quality of the dataset used in themes generation.

Domains considered broadly relevant across all neurological disorders were identified. Under clinician oversight, outputs from each model were compared and annotated for further improvement. Thematic saturation was operationally defined as the stage at which successive prompt iterations produced no substantively new thematic categories following expert review.

### Step 4 – Refining output with human expertise, and selection of preferred LLM

To mitigate AI hallucination or fabrication, outputs were cross-checked against the original dataset of domains. Any domains not present in the source dataset were excluded. LLMs were prompted to provide explanatory rationale for domain classification to facilitate human validation.

The selection criteria included:

1. The ability to accept long prompts sufficient to process the entire domain longlist
2. Generation of outputs that accurately linked domains to relevant core areas
3. Core areas deemed acceptable and meaningful by clinicians

Once the core areas were finalised, a new prompt was designed and tested to link domains to core areas in a tabular form, which was subsequently extracted into Microsoft Excel.

### Step 5- Hierarchical categorisation using clinical expertise, and OMERACT Filter 2.1

**Fig 2:**
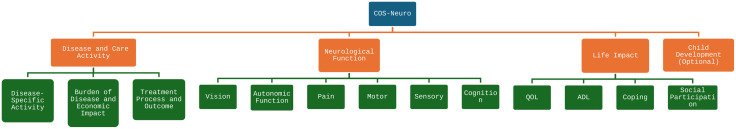
Final conceptual framework for COS-Neuro.

Clinicians worked collaboratively, using the OMERACT Filter 2.1 as a reference framework, to aggregate core areas, into high-level categories (Fig 2) [27]. This included refining the terminology used to describe each core area generated by LLM. Experts were encouraged to draw on their broader medical experience, rather than simply rely on the outcomes from existing neurological COS, to ensure that the resulting framework could be generalised across neurological disorders.

The figure presents the final hierarchical structure of the COS-Neuro framework. Four overarching concepts (orange) were identified: *Disease and Care Activity*, *Neurological Function*, *Life Impact*, and *Child Development* (optional). These concepts are subdivided into 13 core areas (green), which represent key dimensions of outcome assessment across neurological disorders. The framework illustrates the organisation of outcome domains into clinically and conceptually relevant groupings to support outcome selection in neurological research. Abbreviations: QOL, quality of life; ADL, activities of daily living.

## Results

### Step 1 – Dataset gathering

A total of 112 articles on COS relevant to neurological disorders were identified on COMET database, from which 782 core outcomes were extracted. Each study was coded with the COMET ID, and the outcomes were compiled into longlist based on these ID (S1 Supplement 1).

There were 55 neurological disorders with established COS, including condition for which multiple COS exist.

### Step 2 & 3 – Prompt design and initial thematic analysis

The initial prompt was designed to instruct LLMs to perform thematic analysis based on the given longlist of domains. LLMs were specifically asked to provide themes with examples for analysis of the thought process.

Prompt 1:

*Act as a medical researcher. You are given a longlist of core outcome measurement sets on neurology. Please do an inductive thematic analysis of the longlist and summarise maximum of 20 themes. I want domains as examples that linked to the themes. Example of themes would be comprehensive health assessment with symptoms, blood pressure, mental health as domains.*

*Longlist of outcomes*

Feedback on outcome for prompt 1

ChatGPT and Gemini both focused on symptoms, health status, and quality of life, which were subsequently selected as the main themes. The prompt length was too extensive for Llama, necessitating further refinement. The prompt was improved by incorporating common themes reported by both ChatGPT and Gemini as examples and was slightly shortened to accommodate Llama’s input limitations.

The prompt was further edited and improved based on feedback until satisfactory output was achieved in prompt 6. Details on the prompts and their outputs are provided in S2 Supplement 2.

Prompt 6:

*Act as a medical researcher. You are given a longlist of core outcome measurement sets in different neurology disorders. please do an inductive thematic analysis of the given longlist and summarise the main themes suitable for COS in neurology. Please use OMERACT as example with concepts, core areas and domains.*

*Longlist of outcomes*

The outcome generated by ChatGPT is provided in S3 Supplement 3.

### Step 4 – Refining output with human expertise, and selection of preferred LLM

Outputs from ChatGPT, Gemini and Meta were assessed by clinicians based on the criteria detailed in the Methods section. ChatGPT effectively summarised key points relevant in neurology. Gemini followed the OMERACT framework and produced concepts similar to ChatGPT, although its examples were less relevant to neurological disorders. Llama was considered unsuitable for this purpose, as it was unable to process a long list of domains or provide coherent justification for its output. Clinicians therefore agreed to proceed with the outputs of ChatGPT from prompt 6 as the foundation for further COS development. LLMs were used exclusively to assist structural synthesis and categorisation of pre-existing domains rather than to generate novel outcome concepts.

ChatGPT generated 10 themes, which were then established as foundation for COS-Neuro development: Disease Activity/Progression, Physical Functions, Quality of Life and Patient-reported Outcomes, Neurological Symptoms, Health Economics and Health Services, Functional Neurological Disorder (FND) Symptoms, Rehabilitation Outcomes, Perioperative and Surgical Outcomes, Paediatric Neurology Outcomes and Visual and Ocular Outcomes.

ChatGPT was subsequently prompted to classify and tabulate all the relevant domains into these themes. Two rounds of prompt were used to successfully reduce 782 total domains to 218 domains considered relevant to all neurological disorders. The transition from 782 extracted outcome entries to 218 domains followed a reduction process where entries were reviewed for semantic duplication and conceptual overlap. As detailed in S4 supplement 4, the domains were re-categorised or removed based on clinical relevance with panel’s oversight.

Prompt 2:

“*Please analyse ALL the domains given and classify them into the above themes. Provide your answer in a table with two columns. I want the first column to be ALL the longlist of domains given in the first prompt, second column to be the themes. The themes are disease activity/progression, physical functions, Quality of Life and Patient-reported Outcomes, Neurological Symptoms, Health Economics and Health Services, Functional Neurological Disorder (FND) Symptoms, Rehabilitation Outcomes, Perioperative and Surgical Outcomes, Paediatric Neurology Outcomes and Visual and Ocular Outcomes*”

### Step 5 – Hierarchical Categorisation using Clinical Expertise, and OMERACT Filter 2.1

Table 3 outlines the rationale behind each modification to the core areas, including rewording, combining, and further sub-categorisation based on consensus process.

**Table 3:** Improvement of core areas during consensus.

Initial core area	Improved core area	Rationale
Disease activity/progression	Disease-specific activity/progression outcome	The domains in this category focused primarily on seizure activity and control, so disease-specific activity was more appropriate.
Neurological symptoms Visual and ocular outcomes	Pain Sensory function Vision Autonomic functions Cognition Motor functions	Neurological symptoms and visual/ocular outcomes were too broad as core areas, so it was sub-categorising into different neurological functions
Health economics and health service	Burden of disease and economic impact	It was rephrased to healthcare delivery as the domains also consisted of adherence, medication appropriateness, compliance to treatment, education/support which were not limited to economics and services
Quality of life	Quality of life ADL Coping Social participation	QoL had been sub-categorising into QoL, ADL, Coping and social participation as the domains covered a wide range of patient-reported outcomes which were vital in neurological disorders
Rehabilitation outcomes Perioperative and surgical outcomes	Treatment process and outcomes	The two core areas were combined into treatment process and outcomes as they were interconnected and collectively influenced the treatment recovery.
Paediatric neurology outcome	Child development and formation	Paediatric might be easily misunderstood as mixing up with another speciality and the domains such as preschool attendance and performance, engagement in school life were more holistic approach into children’s development
Functional neurological symptom disorder	Removed	FND is a diagnosis rather than a core area.

The final core areas agreed upon by clinicians were disease specific activity, Pain, Sensory function, Vision, Autonomic functions, Cognition, Motor functions, Burden of disease and economic impact, Quality of life, ADL, Coping, Social participation, Treatment process, and Child development and formation.

Once these core areas were established, a final round of prompts was designed to aggregate LLM-generated domains into overarching themes, referred to as ‘main domains’. The aim was to create descriptive terms that would intuitively prompt COS-Neuro users to understand the construct of each area.

Final round of prompt to identify main domains:

*‘Act as medical researcher. You’re developing core outcome measurements in neurological disorders. You’re given the main core areas followed by their domains as text below. I want you to identify the MAIN domains for the core areas.’*

Through this process. 218 domains were further consolidated into 75 domains, as tabulated in Table 4. Candidate domains underwent iterative expert consensus review again during this reduction process. Reduction ceased when further consolidation resulted in loss of clinically meaningful distinction between constructs.

**Table 4:** AI-identified main domains based on clinicians agreed core areas and concept.

Domains	Core area	Concept
Number of new and/or active lesions	Disease-specific activity	Disease and Care Activity
Number of relapses per year (Annual Relapse Rate)		
Switch from relapsing to progressive		
Seizure freedom		
Seizure frequency		
Seizure duration		
Seizure severity		
Hospital admission		
Cost of treatment and healthcare utilization	Burden of disease and economic impact	
Frequency of doctor/clinic visits		
Medication management (adherence, side effects, appropriateness)		
Service provision and related costs		
Hospital admissions and length of stay		
Time to return of full function	Treatment process and outcomes	
Return to work/activity		
Complication and adverse event rates		
Postoperative symptom duration		
Mobility improvement (e.g., walking, standing)		
Surgical outcomes (operative time, incision length, scar)		
Visual field assessment	Vision	Neurological function
Visual functioning		
Eye alignment and movement (ocular motility)		
Functional vision assessment		
Visual fatigue and perception		
Bladder dysfunction and incontinence	Autonomic functions	
Urinary retention		
Faecal incontinence		
Migraine-specific pain	Pain	
Pain due to abnormal sensation		
General pain/discomfort		
Walking and mobility	Motor functions	
Upper extremity function (e.g., grip strength, dexterity)		
Balance and falls		
Muscle strength (e.g., leg, arm)		
Fine and gross motor skills		
Sensation and perception (e.g., paraesthesia, vibratory perception)	Sensory functions	
Sensory function in genital and perineal areas		
Neuroma formation		
Cognitive functioning	Cognition	
Memory		
Executive function		
Concentration		
Health-Related Quality of Life (HRQOL)	Quality of life	Life Impact
Global quality of life		
Treatment-related quality of life		
Life satisfaction		
Global health status		
Fatigue		
Mood swings		
Treatment satisfaction		
Activities of daily living assessment	Activities of daily living (ADL)	
Self-care		
Occupational role functioning		
Ability to do daily activities		
Alertness		
Sleep quality (total sleep, awakenings, daytime sleepiness)		
Self-management	Coping	
Depression and low mood		
Fear of disease progression or complications		
Patient’s ability to understand and respond to communication		
Apathy/indifference		
Patient’s self-esteem and self-worth		
Patient and family perceptions of care quality		
Participation in daily functioning	Social participation	
Social support		
Ability to join in activities with others		
Shared decision-making with healthcare professionals		
Social functioning		
Satisfaction with care and services		
Nutrition, growth, and developmental state	Child Development	
Child physical and emotional health		
Child’s quality of life		
Academic performance and school attendance		
Developmental milestones		
Caregiver quality of life		

The final framework for COS-Neuro included four main concepts as agreed in consensus:

Life impact: including quality of life, ADL, coping, and social participation, representing the social aspects of patients’ life living with neurological disorders.

Neurological function: including vision, autonomic function, pain, motor, sensory and cognition, to establish baseline for patients and characterise disease features.

Disease and care manifestations: sub-divided into disease-specific activity, burden of disease and economic impact, treatment process and outcomes, aimed at evaluating disease progression and therapeutic effectiveness.

Child development and formation: an optional fifth concept, included to address specialised outcomes in paediatric neurological disorders where relevant.

The final framework for COS-Neuro is illustrated in Fig 2.

## Discussion

There is growing awareness and emphasis on using COS in the design and conduct of neurological clinical trials. This is evidenced by their increasing adoption (47% for clinical trials [28]), and ongoing development (48 COS currently in planning or ongoing, according to the COMET database on 26/03/2024)). Nevertheless, many neurological conditions remain unrepresented.

With the assistance of LLMs, particularly ChatGPT, a descriptive thematic synthesis of domains from 112 neurological COS in COMET database was produced. Even though the conceptual decisions were validated through expert consensus, COS-Neuro should be interpreted as a hypothesis-generating, preliminary synthesis: it identifies structural patterns common to existing neurological COS but does not constitute a validated or generalisable standard. A total of four concepts, 13 core areas and 75 domains were finalised following clinical consensus. Given the heterogeneous presentation of neurological diseases, 75 domains were identified to ensure comprehensive coverage of relevant aspects of disease impact. The concept of functional impairment facilitates the evaluation of clinical progression and therapeutic effectiveness. Neurological function domains help establish baselines for patients and understand disease characteristics, while patient-centred outcomes capture the social impact of neurological disorders. Child development and formation were treated as a distinct, optimal concept/core for paediatric neurological disorders.

To our knowledge, COS-Neuro is the first application of AI-assisted thematic synthesis to aggregate existing neurological COS into a descriptive reference framerwork. This represents a methodological contribution to COS development methodology, though its significance relative to established approaches requires empirical evaluation.

A recent study [29] has demonstrated ChatGPT’s ability to enhance the efficiency in thematic analysis by generating meaningful sets of codes and themes. Traditional COS development typically requires manual qualitative data analysis, which is time-consuming and labour-intensive, due to large volumes of textual data that needs to be explored inductively to generate themes and categories [30]. The development of Computer Assisted Qualitative Software (CAQDAS) – such as MAXQDA, NVivo, ATLAS.Ti, and N6- marked an important milestone in qualitative research. These tools offer features such as character-based coding, rich text capabilities, and multimedia support. While CAQDAS improves data transcription efficiency and accelerate analysis, it is underutilised among qualitative health researchers (reported use of only 16%), partially due to the complexity of mastering the platform [31]. Additionally, they are costly, with MAXQDA priced at up to €220 yearly for single user (as of March 2025).

In contrast to traditional CAQDAS tools, LLMs can process large volumes of datasets, identify recurring patterns, generate preliminary thematic summaries rapidly, without requiring specialist software [32]. Most LLMs are free to access and communicate with plain text, making them more intuitive than traditional software. However, effective prompt engineering is pivotal for ensuring relevant, informative, and accurate AI-generated responses. Well-crafted prompts significantly enhance the performance of AI models [23]. In this study, all prompts were carefully designed and under clinical oversight to optimise the outputs. Collaborations between human and LLMs are crucial in improving consistency and reliability of AI-integrated thematic analysis [33].

Importantly, the LLMs used in this study did not generate novel outcome domains but facilitated the organisation and synthesis of existing concepts derived from published COS. Conceptual authority remained with the expert panel, who reviewed, modified, and validated all outputs. The resulting framework should therefore be interpreted as an AI-assisted synthesis rather than an autonomous consensus process. While LLMs enabled scalable aggregation of complex qualitative data, outputs remain sensitive to prompt design and model characteristics, necessitating transparent reporting and human oversight. This framework also identifies and organises outcome domains rather than specifying outcome measurement instruments. Given the cross-condition scope of this study, specifying individual measurement instruments was beyond the intended objectives. Future work should therefore focus on mapping validated measurement tools to the identified domains through dedicated consensus processes aligned with established COS development guidance.

## Advantages and challenges of AI integration

The integration of AI into thematic analysis offered several advantages; however, formal benchmarking against human-only analysis was not conducted and efficiency gains cannot be quantified from this study.

*Efficiency*: AI appeared to facilitate rapid preliminary organisation of domains.
*Consistency*: ChatGPT demonstrated high consistency in thematic groupings, though output stability across sessions was assessed qualitatively rather than through formal intra-rater reliability metrics.
*Feasibility:* This study demonstrates the feasibility of an AI-assisted thematic synthesis approach for aggregating COS domains and offers a methodological template that warrants prospective evaluation in other specialities.

Although AI has been used in medical research [29,34], this is the first reported application of AI-assisted thematic synthesis specifically to aggregate existing COS domains into a descriptive reference framework. Previous studies have demonstrated the utility of ChatGPT as an assistive tool for improving the efficiency of qualitative research, but none have used AI to synthesise themes from a longlist of domains.

Nevertheless. AI integration poses several challenges. A major limitation is the variability in output between different LLMs, even when using the same dataset and prompt. This is due to differences in their underlying architectures and training datasets which may contain bias [32,35]. To mitigate this, we used the same prompt across three models. ChatGPT was selected for final analysis based on its superior consistency and domain relevance, as determined by clinical expertise.

Another key issue is the phenomenon of AI ‘hallucinations’ – where the model generates inaccurate, misleading, or fabricated content. This was addressed by using structured prompt engineering and continuous refinement [36]. A clinician was assigned to lead the process, assess model outputs, and verify the validity of the results. LLMs were also prompted to explain their reasoning, enabling human reviewers to evaluate and, where necessary, correct thematic interpretations. This human-AI collaboration ensured that the final COS-Neuro framework reflect real-world clinical priorities [37].

## Limitations, future development and implementation

While COS-Neuro represents a significant step towards standardisation of outcome selection in neurological trials, several limitations were identified. The domains included in COS-Neuro are bounded entirely by domains present in existing neurological COS in the COMET database. This is not a peripheral limitation but a fundamental feature of the synthesis approach. As a consequence, COS-Neuro cannot serve as a guide for COS developments in conditions without established COS without prospective validation, since domains relevant to those conditions may be systematically absent from the data source. Important outcomes area not represented in existing COS, including sleep and behavioural symptoms such as hallucinations and delusions, should be considered in developing COS, as recognised by consensus among clinical experts given their common presentation in neurological diseases. However, these domains or core areas have not been established as a COS and were therefore not included in the considerations in COS development. COS-Neuro did not conduct formal benchmarking of AI-assisted vs human-only thematic analysis. Claims regarding efficiency and consistency of LLM outputs therefore reflect qualitative observation by the expert panel rather than empirical measurement and should be interpreted accordingly.

Development of COS often involves a broad consensus group with various stakeholders to ensure meaningful and relevant outcomes. However, the development of COS-Neuro was deliberately a streamlined process but considered as appropriate for several reasons: (a) Development of the existing COS in neurological disorders included in this study were inherently resource-intensive, (b) This approach is preliminary and subject to prospective validation and refinement, and (c) Our aim at this stage is to explore feasibility/efficiency. Future iterations of COS-Neuro should incorporate multi-stakeholder engagement to validate and refine the framework in accordance with established COS standards.

The decision to adapt the OMERACT Filter 2.1 as a structural scaffold was pragmatic. Like rheumatological conditions, neurological disorders are complex and heterogeneous. There is considerable overlap, e.g., neurological manifestations of vasculitis or connective tissue diseases. The OMERACT Filter 2.1 was originally developed to improve content validity in outcome selection for Rheumatological trials and has been refined and adopted over time. COS-Neuro does not claim methodological equivalence with OMERACT, which represents decades of iterative, multi-stakeholder consensus development. It was solely used as a pre-existing hierarchical structure (concept > core area > domain) recommended by the COMET Handbook for COS developers [7,8,38], not as a methodological comparator.

Looking ahead, ongoing improvement in AI models could further enhance COS-Neuro. Future iterations may incorporate underrepresented neurological areas, expand domain definitions, and refine thematic structure with additional stakeholder input. As the framework was derived exclusively from neurological COS, its structural properties may reflect discipline-specific research practices and should not be assumed to generalise without further evaluation.

## Practical implication

COS-Neuro may serve as a preliminary structural reference during early stages of COS development discussions for neurological disorders, though it does not substitute for disease-specific, multi-stakeholder consensus and requires prospective validation before use in any specific condition. In the authors’ experience developing a COS for Degenerative Cervical Myelopathy, consolidating and categorising a large and diverse set of outcomes posed substantial challenges [39]. A framework like COS-Neuro would have greatly supported the process.

COS-Neuro may provide a preliminary structural reference for trialists considering outcomes domains in conditions where no disease-specific COS exists but should not be used as a substitute for condition-specific outcome selection guidance. Trialists using COS-Neuro should be aware that its domain coverage is constrained by existing neurological COS and may not reflect the full range of outcomes relevant to their condition. By providing a structured framework, it encourages the use of clinically relevant and patient-centred outcomes, thereby improving consistency and comparability across studies [7,40].

## Conclusion

With the assistance of LLM, COS-Neuro produces a descriptive thematic synthesis of existing neurological COS, organised using the OMERACT Filter 2.1 as a structural scaffold. Of the LLM explored, ChatGPT was considered to be the most effective for this task. The semi-automated approach demonstrates feasibility and may be evaluated in future iterations of COS-Neuro or in other specialties, though transferability should not be assumed without empirical testing.

### Abbreviations

**Table:** 

Acronym	Definition	
COS-Neuro	Core Outcome Sets in Neurological disorders	An ‘COS of COS’ initiative supporting the development of consensus in outcome measurement for neurological disorders.
COS	Core Outcome Sets	An agreed standardised set of outcomes that should be measured and reported, as a minimum, in all clinical trials in specific areas of health or health care.
OMERACT	Outcome Measures in Rheumatoid Arthritis Clinical Trials	www.omeract.org—An initiative supporting the development of consensus in outcome measurement for arthritis.
COMET	Core Outcome Measures in Effectiveness Trials	https://www.comet-initiative.org – An Initiative in development and application of agreed standardised sets of outcomes (COS)
TA	Thematic Analysis	Research method used to identify and interpret patterns or themes in a data set
AI	Artificial Intelligence	Technology that enables computers and machines to simulate human learning, comprehension, problem solving, decision making, creativity and autonomy
LLM	Large Language Models	A type of artificial intelligence algorithm that uses deep learning techniques and massively large data sets to understand, summarize, generate and predict new content
Concept	Overarching idea on important outcomes and guides selection of core areas and domains	
Core area	Distinct aspect of condition or intervention	
Domain	Measurable variables or outcomes from COS	
Outcome	A measurable change or result that can be used to assess the effect of a health care intervention, condition, or disease	
OMERACT Framework	A conceptual framework that helps identify the key areas and domains of health that need to be measured, ensuring a comprehensive approach to outcome measurement	
OMERACT Filter	A tool used to assess and select the appropriate outcome measurement instruments (questionnaires, scales, etc.) for inclusion in the core outcome set.	

## Supporting information

S1 Supplement 1 COMET IDs for included core outcome sets and full longlist of extracted domains. Comprehensive dataset of outcomes extracted from 112 neurological COS used for analysis.
(XLSX)

S2 Supplement 2 Full prompts and outputs from large language models (LLMs). Includes all prompts used across ChatGPT-3.5, Gemini 1.5 Flash, and Llama-2-70B, with corresponding outputs for comparison and reproducibility.
(DOCX)

S3 Supplement 3 Initial AI-generated thematic framework prior to expert refinement. Thematic categorisation produced by ChatGPT before clinician review.
(DOCX)

S4 Supplement 4 Domain re-categorisation and reduction process. Detailed documentation of the iterative process used to consolidate and refine outcome domains, including prompts applied during AI-assisted analysis. This file outlines the methodological steps and decision-making approach used to reduce the dataset to 218 domains aligned with identified thematic groupings.
(XLSX)
