# Supplementary material for: AI-assisted thematic synthesis of existing neurological core outcome sets: A descriptive reference framework (COS-Neuro)
Source: PLoS One. 2026 Jun 22;21(6):e0333864. doi: 10.1371/journal.pone.0333864 (PMC13286209; doi:10.1371/journal.pone.0333864)
Supplement: S2 Supplement 2 — Includes all prompts used across ChatGPT-3.5, Gemini 1.5 Flash, and Llama-2-70B, with corresponding outputs for comparison and reproducibility. (DOCX) [file pone.0333864.s002.docx]

**Prompt 1:**

Act as a medical researcher. You are given a longlist of core outcome measurement sets on neurology. please do an inductive thematic analysis of the longlist and summarise maximum of 20 themes. I want domains as examples that linked to the themes. Example of themes would be comprehensive health assessment with symptoms, blood pressure, mental health as domains.

*ChatGPT*

1. **Clinical Symptoms and Manifestations:**
   - Number of lesions
   - Relapse frequency
   - Switch from relapsing to progressive
   - Cognitive function
   - Walking ability
   - Upper extremity function
   - Balance and falls
   - Visual functioning
   - Bladder dysfunction
   - Fatigue
   - Depression
   - Pain
   - Sensory perception abnormalities
   - Motor function
   - Paraesthesia
   - Neuromuscular signs (e.g., Wartenberg sign, Froment sign)
2. **Health-related Quality of Life:**
   - Comprehensive health assessment
   - Mental health
   - Overall activity level
   - Satisfaction with treatment
   - Self-management abilities
   - Social support
   - Impact on relationships
   - Feeling safe and respected
   - Ability to participate in daily functioning
   - Distress and psychological symptoms
3. **Functional Abilities and Disability:**
   - Participation in daily activities
   - Return to work/function
   - Immobilization duration
   - Motor and sensory impairments
   - Functional limitations (e.g., grip strength, carrying angle)
   - Disability scales and assessments
   - Impact on occupational role functioning
4. **Treatment-related Outcomes:**
   - Operative outcomes (e.g., operative time, complication rate)
   - Postoperative symptoms and duration
   - Rehabilitation outcomes (e.g., return to full function, elbow range of motion)
   - Treatment satisfaction
   - Adverse events and complications
   - Compliance to treatment
5. **Health Economics and Cost-effectiveness:**
   - Health service utilization (e.g., hospital admissions, doctor visits)
   - Cost of medicines and treatment
   - Health-related quality of life measures
   - Economic burden on caregivers
6. **Neurological Functioning and Disease Progression:**
   - Stroke severity and outcomes
   - Body function and structure assessment
   - Disease-specific symptoms (e.g., tremor, muscle weakness)
   - Seizure control and outcomes
   - Cognitive impairment and dementia severity
   - Parkinson's disease-related symptoms
7. **Impact on Daily Living and Social Functioning:**
   - Activities of daily living assessment
   - Social participation and functioning
   - Impact on relationships and social interactions
   - Ability to communicate effectively
   - Emotional well-being and mood
   - Caregiver burden and quality of life
8. **Child-specific Outcomes:**
   - Neurodevelopmental milestones
   - Child pain and physical health state
   - Pediatric quality of life assessments
   - Impact on family well-being
   - Educational and developmental outcomes
9. **Maternal and Pregnancy-related Outcomes:**
   - Seizure control during pregnancy and postpartum
   - Maternal mortality and complications
   - Fetal and neonatal clinical complications
   - Impact on breastfeeding and mode of delivery
10. **Functional Assessments and Measures:**
    - Motor function assessments
    - Gait analysis and mobility assessments
    - Vision and hearing assessments
    - Fine motor abilities
    - Functional 3D skills
    - Communication and speech assessments

*Gemini*

**Patient Function and Ability**

- **Motor Function:** This includes gross motor skills (walking, balance, etc.) and fine motor skills (grip strength, dexterity, etc.). (e.g., Walking, Upper extremity function, Balance/Falls, Grip strength, Power assessment, Elbow flexion test, Return to work duration, Incontinence of Urine, Faecal incontinence, Leg muscle strength, Walking ability, Mobility, Falling/falling over, Balance, Motor Conduction Velocity, etc.)
- **Activities of Daily Living (ADL):** This refers to a person's ability to perform basic tasks for self-care and independent living. (e.g., Activities of daily living assessment, Ability to do daily activities, Ability to perform everyday occupations)
- **Cognitive Function:** This includes memory, attention, problem-solving, and other thinking skills. (e.g., Cognition, Learning, Literacy, Speech and Language, Memory, Concentration, Executive functioning)
- **Communication:** This refers to a person's ability to express themselves and understand others. (e.g., Communication, Patient’s ability to understand and respond to communication from others, Speech, Quality of life questionnaires)
- **Social Participation:** This refers to a person's ability to engage in social activities and relationships. (e.g., Social functioning, Participation, Social support, Importance of relationships, Communication, Feeling safe and secure, Feeling valued and respected by others, Meaningful activities, Social participation, Participating in society)

**Symptoms and Health Status**

- **Pain:** This is a common symptom in many neurological conditions. (e.g., Pain, Neuropathic pain, Migraine Specific Pain, Brief Pain Inventory Facial (BPI-F), Visual Analogue Scale)
- **Fatigue:** This refers to a feeling of tiredness or lack of energy. (e.g., Fatigue, Energy and drive (fatigue), Exercise tolerance)
- **Seizures:** This is a sudden, uncontrolled electrical discharge in the brain that can cause changes in movement, sensation, or consciousness. (e.g., Seizure freedom, Seizure frequency, Seizure duration, Seizure severity)
- **Mental Health:** This includes conditions such as depression, anxiety, and emotional distress. (e.g., Depression, Low Mood and depression, Emotional wellbeing, Mental Health, Anxiety, Emotional distress)
- **Sleep:** This refers to the quality and quantity of sleep a person gets. (e.g., Total time spent asleep at night, Total time spent asleep in 24 hours, Awakenings from sleep, Sleep problems, Sleeping problems)

**Quality of Life**

- **Health-Related Quality of Life (HRQoL):** This is a measure of a person's physical, mental, and social well-being as it is affected by their health condition. (e.g., Health Related quality of life, Global quality of life, Quality of life, Patient Quality of Life)
- **Patient Satisfaction:** This refers to a person's satisfaction with their treatment and care. (e.g., Treatment satisfaction, Patient satisfaction with the intervention)
- **Participation:** This refers to a person's ability to engage in activities that are important to them. (e.g., Participation level in daily functioning, Participation)

**Treatment and Procedures**

- **Surgical Outcomes:** This includes measures of success and complications associated with surgery. (e.g., Operative time, Complication rate, Reoperation rate, Time to return of full function, Immobilization duration)
- **Medication:** This includes measures of effectiveness and side effects of medications. (e.g., Medication appropriateness, Psychotropic drug use, Clinically significant drug interactions, Medication side effects, Adherence)

**Other Important Considerations**

- **Cost-Effectiveness:** This refers to the cost of a treatment compared to its benefits. (e.g., Cost, Health economics/cost–utility)
- **Shared Decision-Making:** This is a process where healthcare professionals and patients work together to make decisions about treatment. (e.g., Shared decision-making between professionals, patients, and family members)
- **Child Development:** This refers to the growth and development of children with neurological conditions. (e.g., Nutrition, growth, and developmental state, Child pain, Child physical health state, Child's quality of life, condition-specific)

*Llama*

Prompt with longlist of domains was too long for llama

**Comments**

Chatgpt and gemini both focused on symptoms/health status and quality of life which could be the main themes. Consider function status or functional ability. Prompt improvement: change examples and shorten the prompt to accommodate llama. Longlist fits in llama but with the prompt included its slightly longer.

**Prompt 2**

Act as a medical researcher. You are given a longlist of core outcome measurement sets. please do an inductive thematic analysis of the longlist and summarise maximum of 20 themes. I want domains to link to the themes. Example of themes could be clinical manifestations or quality of life.

*ChatGPT*

1. **Clinical Manifestations:**
   - Number of new and/or active lesions
   - Number of relapses per year (Annual Relapse Rate)
   - Switch from relapsing to progressive
   - Cognition
   - Walking
   - Upper extremity function
   - Balance/Falls
   - Visual functioning
   - Bladder dysfunction
   - Fatigue
   - Depression
   - Pain
   - 2-Point discrimination
   - Operative time
   - Grip strength
   - Power assessment
   - Postoperative symptom duration
   - Complication rate
   - Reoperation rate
   - Incision length
   - Tourniquet duration
   - Elbow range of motion
   - Tip-to-tip strength
   - Return to work duration
   - Time to return of full function
   - Immobilization duration
   - Ulnar nerve sulcus angle
   - Carrying angle
   - Scar sensitivity
   - Numbness of operating area
   - Sensation
   - Paraesthesia
   - Vibratory perception
   - Motor function
   - Atrophy
   - Ability to hold chopsticks
   - Wartenberg sign
   - Froment sign
   - Claw hand
   - Tinel sign
   - Neuroma formation
   - Return to full activity
   - Elbow instability
   - Flexion contracture
   - Subluxation
   - Elbow flexion test (with or without paresthesia)
   - Symptom recurrence
   - Dropping objects
   - Willingness to repeat treatment
   - Dexterity
   - Esthetics
   - Overall activity level
   - Satisfaction
2. **Quality of Life:**
   - Health-Related quality of life
   - Child's quality of life, condition-specific
   - Global quality of life
   - Occupational role functioning
   - Social functioning
   - Ability to do daily activities
   - Mobility and walking
   - Low Mood and depression
   - General health status
   - Global disability
   - Social participation
   - Ability to communicate
   - Mental Health
   - Sleep quality
   - Emotional well-being
   - Sense of control
   - Participation in society
   - Psychological well-being
3. **Functional Abilities:**
   - Upper limb function
   - Lower limb function
   - Fine motor function
   - Gross motor function
   - Mobility
   - Walking ability
   - Turning in bed
   - Standing up
   - Ability to perform everyday occupations
   - Eating tasks
   - Dressing
   - Hygiene and comfort
4. **Emotional and Psychological Well-being:**
   - Distress around fear of dying or living with severe disability
   - Self-esteem
   - Fears of having a seizure
   - Mood swings
   - Learning capacity
   - Intellectual ability
   - Anxiety
   - Emotional balance
   - Listlessness
   - Aggressiveness
   - Sensitivity to stress
   - Sense of shame
   - Hypersexuality
   - Compulsive repeated actions
   - Frustration
   - Worry
   - Delusions
   - Nightmares
5. **Functional Vision and Eye Health:**
   - Visual acuity
   - Eye alignment position
   - Eye movement—ocular motility assessment
   - Visual field assessment
   - Visual neglect assessment
   - Functional vision assessment
   - Reading assessment
   - Sensitivity to light
   - Ability to watch TV, films or run computer games
   - Ability to focus on near things
   - Ability to track moving objects
   - Ability to recognise visual targets
6. **Caregiver and Family Well-being:**
   - Caregiver quality of life, condition-specific
   - Family Carer Mood
   - Family Carer Burden
   - Family Carer Quality of Life
   - Relevant adults aware of CVI
7. **Health Economics and Cost:**
   - Health economics/cost–utility
   - Cost of medicines
   - Cost of treatment
   - Health-related quality of life
   - Acceptability of the intervention to patients, carers, and stakeholders
   - Patient satisfaction with the intervention
   - Carer satisfaction with the intervention
8. **Social Support and Relationships:**
   - Importance of relationships
   - Feeling valued and respected by others
   - Participate in society
   - Ability to be on visiting terms
   - Social interplay
   - Friendships
   - Engagement in school life
   - Experience of other people’s attitudes towards epilepsy
   - Feelings about having epilepsy

These themes encompass various domains related to clinical outcomes, quality of life, functional abilities, emotional well-being, vision and eye health, caregiver and family well-being, health economics and cost, and social support and relationships. They provide a comprehensive understanding of the diverse aspects considered in core outcome measurement sets across different medical conditions and interventions.

*Gemini*

**Clinical Manifestations:**

- Motor Function (Strength, Coordination, Gait, etc.)
- Sensory Function (Touch, Pain, Vision, etc.)
- Seizures & Epilepsy
- Pain
- Speech & Language
- Cognitive Function (Memory, Learning, etc.)
- Fatigue
- Sleep
- Balance & Falls
- Tremor

**Function & Activity:**

- Activities of Daily Living (ADL)
- Mobility & Walking
- Self-Care & Independence
- Communication
- Participation (Social & Community)
- Work & Productivity

**Health-Related Quality of Life (HRQoL):**

- Overall Well-being & Health Perception
- Emotional Functioning (Mood, Anxiety, Depression)
- Social Functioning
- Patient Satisfaction

**Other:**

- Complications & Adverse Events
- Mortality & Survival
- Resource Utilization (Costs, Hospital Admissions)
- Psychological Distress & Fear
- Aesthetics & Body Image
- Medication Side Effects & Adherence

**Note:** These themes are grouped into broader categories for easier navigation. Some outcomes may fit into multiple themes.

*Llama*

Prompt was too long for llama

**Comment**

Llama doesn’t seem like a viable model to use. Consider other LLM to compare. Gemini has much shorter and focused themes compared to ChatGPT. Remove example in the next prompt.

**Prompt 3**

Act as a medical researcher. You are given a longlist of core outcome measurement sets. please do an inductive thematic analysis of the longlist and summarise maximum of 20 themes. I want domains to link to the themes.

*ChatGPT*

1. **Physical Functioning and Mobility**:
   - Walking ability
   - Upper extremity function
   - Balance/Falls
   - Muscle strength
   - Ability to perform everyday activities
2. **Quality of Life and Well-being**:
   - Health-related quality of life
   - Overall activity level
   - Satisfaction with treatment
   - Emotional well-being
   - Social functioning
3. **Symptom Management**:
   - Pain intensity and frequency
   - Fatigue
   - Depression
   - Anxiety
   - Sleep quality and disturbances
4. **Functional Outcomes**:
   - Cognitive functioning
   - Communication abilities
   - Activities of daily living assessment
   - Fine motor skills and dexterity
5. **Complications and Adverse Events**:
   - Complication rate
   - Reoperation rate
   - Adverse events related to treatment or condition
6. **Patient Satisfaction and Experience**:
   - Treatment satisfaction
   - Shared decision-making between patients, caregivers, and healthcare professionals
   - Perception of being valued and respected as a person
7. **Neurological Functioning**:
   - Sensory perception
   - Motor function
   - Presence of neurological symptoms (e.g., paraesthesia, neuropathic pain)
8. **Economic and Resource Utilization**:
   - Health economics/cost–utility
   - Hospital admissions
   - Cost of medicines/treatment
9. **Specific Condition-related Outcomes**:
   - Disease-specific symptoms and complications
   - Progression of the condition
   - Seizure control (for epilepsy)
   - Neurological status (for traumatic brain injury, Parkinson's disease, etc.)
10. **Family and Caregiver Outcomes**:
    - Caregiver burden
    - Impact on family well-being and quality of life
11. **Functional Neurological Disorder (FND) Symptoms**:
    - Core FND symptoms
    - Psychological symptoms
    - Life impact
12. **Pediatric Outcomes**:
    - Developmental milestones
    - Quality of life in children and caregivers
    - Growth and nutrition
13. **Ocular Outcomes**:
    - Visual functioning
    - Eye movement and alignment
    - Functional vision assessment
14. **Recovery and Rehabilitation**:
    - Return to work duration
    - Time to return to full function
    - Rehabilitation outcomes and progress
15. **Communication and Social Participation**:
    - Ability to communicate effectively
    - Participation in social activities and society
16. **Disease-specific Health Parameters**:
    - Disease severity
    - Disease-specific disability scales
    - Seizure frequency and severity
17. **Patient-reported Outcomes**:
    - Patient mood
    - Patient activities of daily living
    - Patient behavior and global function
18. **Treatment-specific Outcomes**:
    - Treatment adherence
    - Response to treatment
    - Treatment-related complications
19. **Maternal and Neonatal Outcomes**:
    - Pregnancy-related complications
    - Neonatal health and development
20. **Surgical and Procedural Outcomes**:
    - Operative time
    - Surgical site infection
    - Functional outcomes post-surgery

*Gemini*

**1. Physical Function:** This theme includes outcomes related to mobility, walking, balance, upper extremity function, grip strength, and activities of daily living (ADL).

**2. Pain:** This theme captures the intensity, location, and impact of pain on patients' lives.

**3. Neurological Function:** This theme encompasses outcomes related to sensation, motor function, reflexes, and nerve function.

**4. Quality of Life:** This theme assesses the overall impact of a condition or treatment on a patient's well-being, including physical, mental, social, and emotional aspects.

**5. Mental Health:** This theme includes outcomes related to depression, anxiety, mood, and emotional well-being.

**6. Cognitive Function:** This theme assesses a patient's ability to think, learn, and remember.

**7. Fatigue:** This theme captures the level of tiredness and its impact on daily activities.

**8. Complications:** This theme identifies any adverse events or unintended consequences of a treatment or condition.

**9. Return to Function:** This theme assesses how quickly a patient can resume their usual activities after treatment or diagnosis.

**10. Patient Satisfaction:** This theme captures a patient's level of satisfaction with a treatment or intervention.

**11. Social Function:** This theme assesses a patient's ability to participate in social activities and relationships.

**12. Self-Care:** This theme assesses a patient's ability to perform basic tasks for themselves, such as bathing and dressing.

**13. Sleep:** This theme assesses the quality and quantity of sleep a patient gets.

**14. Communication:** This theme assesses a patient's ability to understand and express themselves.

**15. Cost-Effectiveness:** This theme examines the balance between the cost of a treatment and its benefits.

**16. Treatment Burden:** This theme assesses the impact of treatment on a patient's daily life, such as time commitment and side effects.

**17. Patient-Reported Outcomes (PROs):** This theme emphasizes outcomes directly reported by patients about their experiences and perceptions.

**18. Disease Specificity:** This theme includes outcomes specific to a particular disease or condition.

**19. Safety:** This theme assesses the potential for harm associated with a treatment or intervention.

**20. Functional Outcomes:** This theme focuses on a patient's ability to perform specific tasks and activities relevant to their daily life.

**Domains:**

These themes can be further categorized into broader domains such as:

- Musculoskeletal
- Neurological
- Psychological
- Social
- Functional
- Economic

By analyzing core outcome measurement sets with a thematic approach, researchers can identify key areas to be measured in clinical trials and understand the most important outcomes for patients.

*Llama*

Prompt was too long

**Comments**

Without providing examples in prompt, both LLMs give quite a broad selection of themes but with a few similarities. Looking at the trends from previous prompts, important themes would be quality of life, functional outcomes, clinical manifestations would be the main ones we should focus on (as highlighted in red). However, for the prompts to be more effective, we should write prompt that link domains to specific themes. At this stage, Llama would not be a suitable choice of LLM to use.

**Prompt 4**

Act as medical researcher. Please conduct inductive thematic analysis based on the longlist of domains given below. The longlist with domains provided below are extracted from different core outcome sets paper related to neurology. I want the domains to link to the themes with explanations. Please give me 10 main themes based on the domains given. Examples of themes are clinical manifestations, quality of life and functional outcomes.

*ChatGPT*

1. **Functional Status and Mobility**
   - Includes domains such as walking ability, upper extremity function, balance/falls, motor function, fine motor ability, standing up, turning in bed, and posture.
   - Reflects the patient's ability to perform daily activities independently and move around effectively.
2. **Quality of Life and Well-being**
   - Encompasses domains related to health-related quality of life, social participation, activities of daily living assessment, participation level in daily functioning, psychological well-being, emotional functioning, mood, self-esteem, and self-care.
   - Focuses on the overall impact of the condition on the patient's life satisfaction, emotional state, and social interactions.
3. **Pain Management**
   - Encompasses domains related to pain intensity, frequency, and duration, as well as neuropathic pain, ache, and headache.
   - Addresses the patient's experience of pain and the effectiveness of pain management strategies.
4. **Cognitive Function and Mental Health**
   - Includes domains such as cognition, depression, anxiety, memory, executive functioning, insight, judgement, concentration, and intellectual ability.
   - Assesses cognitive abilities, mental health status, and emotional regulation in patients with neurological conditions.
5. **Treatment Outcomes and Adverse Events**
   - Encompasses domains related to treatment satisfaction, complications, reoperation rate, adverse events, medication side effects, adherence, and mortality.
   - Evaluates the effectiveness and safety of treatments, as well as their impact on patient outcomes.
6. **Neurological Symptoms and Functioning**
   - Includes domains such as sensation, motor nerve conduction velocity, muscle weakness, tremor, tingling sensation, cramps, speech problems, and vision.
   - Focuses on specific neurological symptoms and their impact on daily functioning.
7. **Social Support and Caregiver Burden**
   - Encompasses domains related to caregiver quality of life, family carer burden, family well-being, and social support.
   - Addresses the impact of the condition on caregivers and the importance of social support networks.
8. **Economic and Health Services Impact**
   - Includes domains such as health economics/cost–utility, hospital admissions, health parameters, medication appropriateness, and health-related quality of life.
   - Examines the economic burden of the condition and the efficiency of healthcare services in managing it.
9. **Patient Perception and Communication**
   - Encompasses domains related to patient satisfaction, shared decision-making, communication, and distress.
   - Focuses on patient-centered care, communication between healthcare professionals and patients, and patient perceptions of their condition and treatment.
10. **Developmental and Paediatric Outcomes**
    - Includes domains such as developmental milestones, neurodevelopment, growth, and pediatric-specific quality of life measures.
    - Addresses the unique needs and outcomes of pediatric patients with neurological conditions, considering their developmental stages and long-term implications.

*Gemini*

1. **Impairment and Function:** This theme covers limitations in a person's ability to perform activities due to neurological conditions.
   - Domains: Number of new and/or active lesions, Walking, Upper extremity function, Balance/Falls, Visual functioning, Bladder dysfunction, Ulnar nerve sensory impairment, Median nerve motor impairment, Peroneal nerve impairment, Hand grip impairment, Hand pinch impairment, Motor Conduction Velocity, Distal Latency, Compound Muscle Action Potential, Sensory Conduction Velocity, Sensory Action Potential, Rigidity, Posture, Arm swing, Hyperkinesia, Walking ability, Tremor, Muscle weakness, Turning in bed, Slow movements, Writing difficulties, Fine motor ability, Freezing, Mobility, Motor/mental fluctuations, Passive External Rotation (in adduction), Passive abduction, Passive Elbow extension, Active External Rotation (in adduction), Active External Rotation (in abduction), Active Abduction, Active Elbow Flexion, Active Elbow Extension, Active Wrist Extension, Active Finger Flexion, Active Finger Extension, Gait analysis (kinematics with or without kinetics), Gait velocity, Gait Profile Score, Gait Deviation Index, Gillette Gait Index
2. **Pain:** This theme focuses on the experience of pain associated with neurological conditions.
   - Domains: Pain, Neuropathic pain, Brief Pain Inventory Facial (BPI-F), Visual Analogue Scale, Barrow Neurology Institute Pain Scale, Penn Facial Pain Scale-Revised, Trigeminal Neuralgia Quality of Life Score
3. **Quality of Life:** This theme encompasses a person's overall well-being and satisfaction with life in the context of their neurological condition.
   - Domains: Health Related quality of life, Global quality of life, Patient Quality of Life, SF-36, EuroQol, Boulton’s quality of life measure, Vickrey’s quality of life measure, Quality of life and disability measures, Amsterdam Linear Disability Status Scale, INCAT overall 12-point disability scale, Charcot-Marie-Tooth neuropathy score, Overall disability sumscore, Health-related quality of life, Trigeminal Neuralgia Quality of Life Score
4. **Mental Health:** This theme includes emotional and psychological aspects of neurological conditions.
   - Domains: Depression, Low Mood and depression, Mood swings, Emotional well-being, Anxiety, Emotional distress, Delirium occurrence and reoccurrence, Delirium severity, Delirium duration, Behaviour/anxiety/depression/PTSD
5. **Cognitive Function:** This theme refers to a person's ability to think, learn, and remember.
   - Domains: Cognition, Cognitive impairment, Learning, Literacy, Memory, Concentration, Executive functioning, Seizure frequency, Seizure severity, EEG response, learning capacity (difficult to learn new things), Short-term memory, Intellectual ability, Insight, Judgement
6. **Social Functioning:** This theme covers a person's ability to interact with others and participate in social activities.
   - Domains: Social participation, social functioning, Communication, Social support, Importance of relationships, Communication, feeling safe and secure, feeling valued and respected by others, Participating in society, Socializing, Community/social life and relationships
7. **Activities of Daily Living (ADL):** This theme refers to the basic tasks of self-care that people need to perform in everyday life.
   - Domains: Self-care, Independence, Activities of daily living assessment, Patient Activities of Daily Living /Instrumental Activities of Daily Living, Self-care/fine motor skills, Toileting
8. **Mobility:** This theme focuses on a person's ability to move around.
   - Domains: Mobility, Walking, walking ability, Falling/falling over, Balance, Gait analysis (kinematics with or without kinetics), Gait velocity, Gait Profile Score, Gait Deviation Index, Gillette Gait Index
9. **Seizure Control:** This theme is relevant to epilepsy and focuses on controlling or preventing seizures.
   - Domains: Seizure freedom, Seizure frequency, Seizure duration, Clinical response (no clinical evidence of spasm), EEG response (no longer indicates West Syndrome)
10. **Fatigue:** This theme refers to a feeling of tiredness or lack of energy.
    - Domains: Fatigue, Energy and drive functions, Tiredness/fatigue

**Comments**

Not a great prompt. Both LLMs give different outcomes. But explanation would be good to capture reasoning behind. Stick to prompt 3 but ask for three main themes and example from Omeract with concepts, core areas and domain

**Prompt 5**

Act as a medical researcher. You are given a longlist of core outcome measurement sets. please do an inductive thematic analysis of the longlist and summarise the main themes. Please use OMERACT as example with concepts, core areas and domains based on the longlist of outcome given.

*ChatGPT*

After conducting an inductive thematic analysis of the longlist of core outcome measurement sets, several main themes emerge across various medical conditions and interventions. These themes encompass a wide range of aspects related to patient health, well-being, functional abilities, and treatment outcomes. One illustrative example that captures these themes is OMERACT (Outcome Measures in Rheumatology), which focuses on rheumatology-related outcomes. Here's how the concepts, core areas, and domains from the longlist can be mapped onto the OMERACT framework:

1. **Disease Activity and Progression**:
   - Number of new and/or active lesions
   - Number of relapses per year (Annual Relapse Rate)
   - Switch from relapsing to progressive
2. **Physical Function and Mobility**:
   - Walking
   - Upper extremity function
   - Balance/Falls
   - Grip strength
   - Ability to hold chopsticks
   - Elbow range of motion
   - Flexion contracture
3. **Cognitive Function and Emotional Well-being**:
   - Cognition
   - Depression
   - Fatigue
   - Anxiety
   - Psychological symptoms
   - Apathy/indifference
   - Alertness
4. **Quality of Life and Participation**:
   - Health Related quality of life
   - Participation level in daily functioning
   - Satisfaction
   - Activities of daily living assessment
   - Social functioning
   - Occupational role functioning
   - Overall activity level
5. **Patient Experience and Perception**:
   - Distress around fear of dying or living with severe disability
   - Feeling treated and valued as a person
   - Treatment satisfaction
   - Patient and family perceptions of care appropriateness
   - Shared decision-making between professionals, patients, and family members
6. **Health Economics and Cost-Utility**:
   - Cost
   - Health economics/cost–utility
   - Cost of medicines
   - Caregiver burden
7. **Clinical and Functional Outcomes**:
   - Motor function
   - Pain
   - Sensation
   - Muscle strength
   - Range of motion
   - Postoperative symptom duration
   - Complication rate
   - Reoperation rate
8. **Neurological and Sensory Function**:
   - Visual functioning
   - Bladder dysfunction
   - Paraesthesia
   - Vibratory perception
   - Neuroma formation
   - Tinel sign
   - Ulnar nerve sensory impairment
   - Sensory Conduction Velocity
9. **Patient and Caregiver-reported Outcomes**:
   - Treatment satisfaction
   - Adherence to treatment
   - Impact on daily life
   - Caregiver quality of life
10. **Long-term and Overall Health Status**:
    - Global quality of life
    - Overall disability
    - Survival
    - General health status

**Comment**

Misunderstood as domains from rheumatology. Add neurology onto the next prompt.

Prompt 6:

Act as a medical researcher. You are given a longlist of core outcome measurement sets in different neurology disorders. please do an inductive thematic analysis of the given longlist and summarise the main themes suitable for COS in neurology. Please use OMERACT as example with concepts, core areas and domains.

*ChatGPT*

To conduct an inductive thematic analysis of the given long list of core outcome measurement sets (COMS) in different neurology disorders, we will first categorize the provided outcomes into broader themes and then summarize these themes suitable for COS in neurology. As an example, we'll refer to OMERACT (Outcome Measures in Rheumatology) which provides a structured framework for core areas and domains.

1. **Disease Activity/Progression:**
   - Number of new and/or active lesions
   - Number of relapses per year (Annual Relapse Rate)
   - Switch from relapsing to progressive
   - Cognitive function
   - Motor function
   - Seizure frequency/severity/duration
2. **Physical Function:**
   - Walking ability
   - Upper extremity function
   - Grip strength
   - Balance/Falls
   - Fine motor ability
   - Ability to perform everyday activities (Activities of Daily Living)
3. **Quality of Life and Patient-reported Outcomes:**
   - Health-related quality of life
   - Treatment satisfaction
   - Patient and family perceptions of care
   - Patient mood (Depression, Anxiety)
   - Self-esteem
   - Social functioning and participation
   - Satisfaction with treatment and outcomes
   - Overall activity level
   - Fatigue
   - Pain
   - Sleep quality
   - Emotional well-being
4. **Neurological Symptoms:**
   - Visual functioning
   - Bladder and bowel dysfunction
   - Sensory impairments (paraesthesia, vibration perception)
   - Neuropsychological symptoms
   - Psychological symptoms (Anxiety, Depression)
   - Cognitive functioning
5. **Health Economics and Health Services:**
   - Health economics/cost–utility
   - Hospital admissions
   - Caregiver burden
   - Cost of treatment and care
   - Adherence to treatment
6. **Functional Neurological Disorder (FND) Symptoms:**
   - Core FND symptoms
   - Other physical and psychological symptoms
   - Life impact
   - Distress and coping mechanisms
7. **Rehabilitation Outcomes:**
   - Return to work duration
   - Time to return of full function
   - Mobility and walking improvements
   - Rehabilitation complications and adverse events
   - Ability to participate in daily activities
8. **Perioperative and Surgical Outcomes:**
   - Operative time
   - Postoperative symptom duration
   - Complication rate
   - Reoperation rate
   - Scar sensitivity
   - Numbness and sensation in the operating area
   - Post-surgery motor function and recovery
9. **Pediatric Neurology Outcomes:**
   - Child pain
   - Developmental milestones
   - Quality of life in children and caregivers
   - Pediatric-specific neurological symptoms and disabilities
   - School attendance and performance
10. **Visual and Ocular Outcomes:**
    - Visual acuity
    - Ocular motility and alignment
    - Visual field assessment
    - Functional vision and reading assessment
    - Quality of life related to vision and ocular symptoms

These themes provide a comprehensive overview of the core outcome areas relevant to neurological disorders. COS in neurology should aim to capture these diverse aspects to ensure a holistic assessment of disease impact, treatment efficacy, and patient well-being

*Gemini*

## Core Outcome Measurement Sets for Neurological Disorders following OMERACT principles

**OMERACT (Outcome Measures in Rheumatology)** provides a framework for evaluating outcome measurement instruments in rheumatology. Here's an analysis of the provided longlist following OMERACT principles:

**Concepts:**

- **Body Functions & Structures:** This captures anatomical and physiological impairments like weakness, spasticity, tremor, vision, and hearing loss. (e.g., Grip Strength, Visual Acuity)
- **Activities & Participation:** This assesses a person's ability to perform daily tasks and participate in social life. (e.g., Activities of Daily Living, Walking Ability)
- **Health Related Quality of Life (HRQoL):** This measures the impact of the neurological condition on a person's well-being and perception of health. (e.g., SF-36, EuroQol)

**Core Areas:**

- **Impairment:** Measures focusing on body functions and structures. (e.g., Motor Conduction Velocity, Ulnar Nerve Sensory Impairment)
- **Activity Limitation:** Measures focusing on limitations in performing daily tasks. (e.g., Timed Up and Go, Gait analysis)
- **Participation Restriction:** Measures focusing on limitations in social participation. (e.g., Community/social life and relationships)
- **HRQoL:** Measures the overall impact on a person's well-being.

**Domains:**

- **Musculoskeletal:** This focuses on muscles, bones, and joints. (e.g., Grip Strength, Gait analysis)
- **Neurological:** This focuses on the nervous system and its functions. (e.g., Seizure Frequency, Tremor)
- **Cognitive:** This focuses on thinking, memory, and learning. (e.g., Learning and accessing books or screens, Cognitive Function)
- **Psychological:** This focuses on emotions, mood, and behavior. (e.g., Depression, Anxiety)
- **Social:** This focuses on social interaction and relationships. (e.g., Family well-being, Community Participation)

**Examples:**

- **Stroke:** Core outcome sets for stroke might focus on body functions (muscle weakness, paralysis), activity limitations (walking ability, self-care), participation restrictions (returning to work, social interaction), and HRQoL. Specific outcome measures could include grip strength, modified Rankin Scale, and SF-36.
- **Parkinson's Disease (PD):** Core outcome sets for PD might focus on motor function (tremor, rigidity), activities of daily living (dressing, eating), HRQoL, and medication side effects. Specific outcome measures could include the Unified Parkinson's Disease Rating Scale (UPDRS) and PD-related quality of life questionnaires.

**Conclusion:**

This analysis highlights the importance of using a comprehensive approach to outcome measurement in neurology, considering body functions, activity limitations, participation restrictions, and HRQoL. By following OMERACT principles, researchers can develop core outcome sets specific to each neurological condition, facilitating more meaningful clinical trials and improved patient care.

**Comment**

ChatGPT had summarised all the important points that were relevant in neurology and Gemini had followed omeract pattern and has quite similar concepts to ChatGPT but the example isn’t quite useful. Decision to adapt ChatGPT as LLM of choice to proceed.
