# Supplementary material for: AI-assisted thematic synthesis of existing neurological core outcome sets: A descriptive reference framework (COS-Neuro)
Source: PLoS One. 2026 Jun 22;21(6):e0333864. doi: 10.1371/journal.pone.0333864 (PMC13286209; doi:10.1371/journal.pone.0333864)
Supplement: S3 Supplement 3 — Thematic categorisation produced by ChatGPT before clinician review. (DOCX) [file pone.0333864.s003.docx]

ChatGPT output

“To conduct an inductive thematic analysis of the given long list of core outcome sets (COS) in different neurology disorders, we will first categorize the provided outcomes into broader themes and then summarize these themes suitable for COS in neurology. As an example, we'll refer to OMERACT (Outcome Measures in Rheumatology) which provides a structured framework for core areas and domains.”

| **Themes** | **Domains** |
| --- | --- |
| Disease activity/progression | Number of new and/or active lesions |
|  | Number of relapses per year (Annual Relapse Rate) |
|  | Switch from relapsing to progressive |
|  | Cognitive function |
|  | Motor function |
|  | Seizure frequency/severity/duration |
| Physical Function | Walking ability |
|  | Upper extremity function |
|  | Grip strength |
|  | Balance/Falls |
|  | Fine motor ability |
|  | Ability to perform everyday activities (Activities of Daily Living) |
| Quality of Life and Patient-reported Outcomes | Health-related quality of life |
|  | Treatment satisfaction |
|  | Patient and family perceptions of care |
|  | Patient mood (Depression, Anxiety) |
|  | Self-esteem |
|  | Social functioning and participation |
|  | Satisfaction with treatment and outcomes |
|  | Overall activity level |
|  | Fatigue |
|  | Pain |
|  | Sleep quality |
|  | Emotional well-being |
| Neurological Symptoms | Visual functioning |
|  | Bladder and bowel dysfunction |
|  | Sensory impairments (paraesthesia, vibration perception) |
|  | Neuropsychological symptoms |
|  | Psychological symptoms (Anxiety, Depression) |
|  | Cognitive functioning |
| Health Economics and Health Services | Health economics/cost–utility |
|  | Hospital admissions |
|  | Caregiver burden |
|  | Cost of treatment and care |
|  | Adherence to treatment |
| Functional Neurological Disorder (FND) Symptoms | Core FND symptoms |
|  | Other physical and psychological symptoms |
|  | Life impact |
|  | Distress and coping mechanisms |
| Rehabilitation Outcomes | Return to work duration |
|  | Time to return of full function |
|  | Mobility and walking improvements |
|  | Rehabilitation complications and adverse events |
|  | Ability to participate in daily activities |
| Perioperative and Surgical Outcomes | Operative time |
|  | Postoperative symptom duration |
|  | Complication rate |
|  | Reoperation rate |
|  | Scar sensitivity |
|  | Numbness and sensation in the operating area |
|  | Post-surgery motor function and recovery |
| Paediatric Neurology Outcomes | Child pain |
|  | Developmental milestones |
|  | Quality of life in children and caregivers |
|  | Paediatric-specific neurological symptoms and disabilities |
|  | School attendance and performance |
| Visual and Ocular Outcomes | Visual acuity |
|  | Ocular motility and alignment |
|  | Visual field assessment |
|  | Functional vision and reading assessment |
|  | Quality of life related to vision and ocular symptoms |
| These themes provide a comprehensive overview of the core outcome areas relevant to neurological disorders. COS in neurology should aim to capture these diverse aspects to ensure a holistic assessment of disease impact, treatment efficacy, and patient well-being. | |
